# SP600125 has a remarkable anticancer potential against undifferentiated thyroid cancer through selective action on ROCK and p53 pathways

**DOI:** 10.18632/oncotarget.5799

**Published:** 2015-09-22

**Authors:** Elisa Stellaria Grassi, Valeria Vezzoli, Irene Negri, Árpád Lábadi, Laura Fugazzola, Giovanni Vitale, Luca Persani

**Affiliations:** ^1^ DISCCO, Department of Clinical Sciences and Community Health, University of Milan, Milan, Italy; ^2^ Laboratory of Endocrine and Metabolic Research, Istituto Auxologico Italiano IRCCS, Cusano Milanino, Italy; ^3^ Department of Laboratory Medicine, University of Pécs, Pécs, Hungary; ^4^ Department of Pathophysiology and Transplantation, University of Milan, Milan, Italy; ^5^ Endocrine Unit-Fondazione IRCCS Ca’ Granda, Milan, Italy; ^6^ Division of Endocrine and Metabolic Diseases, Istituto Auxologico Italiano IRCCS, Milan, Italy; ^7^ Current address: IRIBHM, Institute of Interdisciplinary Research in Molecular Human Biology, Université Libre de Bruxelles, Brussels, Belgium

**Keywords:** thyroid cancer, SP600125, p53, ROCK, mitotic catastrophe

## Abstract

Thyroid cancer is the most common endocrine malignancy with increasing incidence worldwide.

The majority of thyroid cancer cases are well differentiated with favorable outcome. However, undifferentiated thyroid cancers are one of the most lethal human malignancies because of their invasiveness, metastatization and refractoriness even to the most recently developed therapies.

In this study we show for the first time a significant hyperactivation of ROCK/HDAC6 pathway in thyroid cancer tissues, and its negative correlation with p53 DNA binding ability.

We demonstrate that a small compound, SP600125 (SP), is able to induce cell death selectively in undifferentiated thyroid cancer cell lines by specifically acting on the pathogenic pathways of cancer development. In detail, SP acts on the ROCK/HDAC6 pathway involved in dedifferentiation and invasiveness of undifferentiated human cancers, by restoring its physiological activity level. As main consequence, cancer cell migration is inhibited and, at the same time, cell death is induced through the mitotic catastrophe. Moreover, SP exerts a preferential action on the mutant p53 by increasing its DNA binding ability. In TP53-mutant cells that survive mitotic catastrophe this process results in p21 induction and eventually lead to premature senescence. In conclusion, SP has been proved to be able to simultaneously block cell replication and migration, the two main processes involved in cancer development and dissemination, making it an ideal candidate for developing new drugs against anaplastic thyroid cancer.

## INTRODUCTION

Thyroid cancer is the most common endocrine malignancy and its incidence is constantly increasing worldwide [1-3]. Although well-differentiated cancers (WDTCs) with good prognosis and favourable outcome constitute the large majority of thyr*oid can*cers, the poorly differentiated (PDTCs) and anaplastic (ATCs) ones account for a significant portion of the morbidity and mortality due to thyroid cancer [1, 4].

The pathogenesis of thyroid cancers is characterized by sequential accumulation of different genetic alterations. In particular, development of either PDTCs or ATCs results from genomic instability and accumulation of multiple aberrations leading to cell cycle deregulation and acquisition of anchorage-independent growth. These alterations are known to be responsible for the aggressive clinical features of these lethal tumors that are resistant to radioiodine, to conventional chemotherapy and external radiation, and even to tyrosine-kinase inhibitors (TKI) [1, 5-7].

In the last decades, numerous alterations in proteins involved in thyroid cancer dedifferentiation process, including RAS, ALK, β-catenin and p53, have been identified [8-10]. In particular, mutations in β-catenin and p53 genes are almost absent in WDTCs but can be found respectively in up to 60% and 80% of ATCs [5].

In the present study, we explore the efficacy of a small compound, SP600125 (SP) in counteracting the aggressive tumorigenic behavior of undifferentiated thyroid cancer cells.

SP is a reversible ATP-competitive multikinase inhibitor with recently discovered intriguing anticancer properties. It was shown that SP modifies cell cycle progression and causes endoreduplication with preferential activity against p53 null cells [11-13], induces p53-independent apoptosis and cell cycle arrest [14-15] and increases cell sensitivity to various anti-proliferative drugs [16-17]. All of the reported mechanisms of action make SP an ideal candidate for developing new therapies against aggressive cancers, such as ATCs. However, most of the previously described SP actions imply a lack of target specificity that would undermine any further clinical development as anticancer treatment.

Here we describe for the first time a remarkable SP selectivity against cancer cells harboring *TP53* point mutations and concomitant hyper-activation of Rho associated kinase (ROCK). We demonstrate that SP induces cell death and migration inhibition through the activation of mutant p53 and concomitant ROCK/HDAC6 pathway inhibition.

Moreover we provide new insights about the relationship between p53 inactivation and ROCK hyperactivation in thyroid cancer.

It has recently been reported tha loss of p53 results in hyperactivation of RhoA/ROCK pathway and this can lead to increased invasiveness [18-20]. In agreement with these findings our results shows for the first time an increase of ROCK activity in PTDCs and a strong inverse correlation between p53 DNA binding ability and ROCK activity in thyroid cancer tissues and cell lines.

The relevance and the potential impact of our findings are underlined by recent studies emphasizing the importance of p53 targeted therapy both in preclinical and clinical settings [21-23]. Furthermore they highlight the role of ROCK kinases in cancer cell invasion, in accordance with the recent clinical trials with ROCK-targeting compounds [24-26].

## RESULTS

### SP inhibits cell proliferation in a p53 dependent way

**Figure 1:**
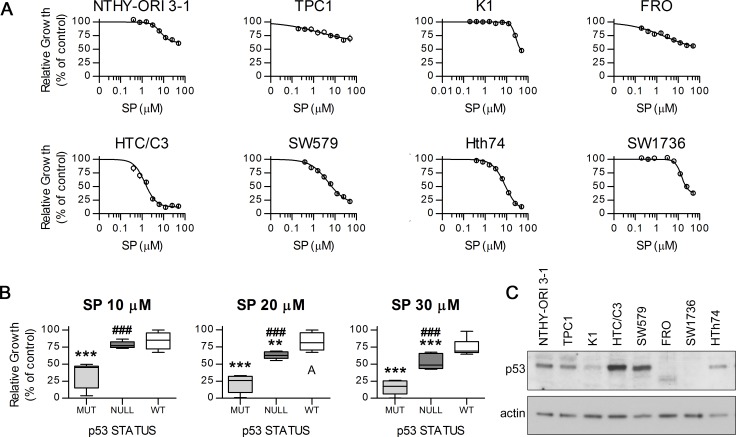
Antiproliferative effect of SP on distinct thyroid cancer cell lines with different *TP53* status Cell lines were exposed to different concentrations of SP or equal amount of DMSO for 96 hours and growth inhibition was assessed with MTT assay. **A.** Growth curves of the cell lines showing different degrees of inhibition. **B.** SP effects on the cell lines grouped by p53 status. **C.** Western blot confirming the differential expression of p53 in our cell lines; actin was used as loading control. MUT, p53 missense mutant, HTC/C3, Hth74 and SW579 cells; NULL, p53 pseudo-null, SW1736 and FRO cells; WT, p53 wild type, NTHY-ORI 3-1, TPC1 and K1 cells. ***p < 0.001, **p < 0.01 *vs*. WT, ###*p* < 0.001 *vs*. MUT.

SP anti-proliferative effects were assessed *in vitro* on a normal thyroid derived cell line and seven thyroid cancer cell lines (Figure 1A) with genetic alterations typical of different thyroid cancer subtypes such as BRAF^V600E^ variant, *RET/PTC* translocation, PI3K pathway hyperactivation and *TP53* point mutations (see Supplemental Material and Methods). In accordance with previously published data [13], analysis of growth inhibition curves revealed that, at concentrations equal or higher than 30 μM, SP is highly effective against poorly differentiated cells that lack p53 activity (Figure 1B). Moreover we show for the first time a preferential activity of low dose SP treatment on cells with missense inactivating *TP53* alterations versus *TP53*-null ones. The selectivity against p53 mutant cells became even more apparent at concentrations of 10 μM or lower (Figure 1B). The relative amounts of p53 protein in our cell lines was confirmed by western blot analysis (Figure 1C). Importantly, no significant association between BRAF or PI3K pathways hyperactivation and SP antiproliferative effects was found (Supplementary Table 1).

The concentrations of 10 and 20 μM were thus chosen for further investigations.

### SP induces premature senescence through the p53/p21 pathway

Up to date, there are contrasting reports about p53 involvement in the SP mechanism of action [11, 13-15, 27]. To elucidate this point, three cell lines representing different *TP53* status were chosen for further examination: the *TP53* wild-type TPC1, the *TP53* p.P152L mutant HTC/C3, and the p53 pseudo-null SW1736.

**Figure 2:**
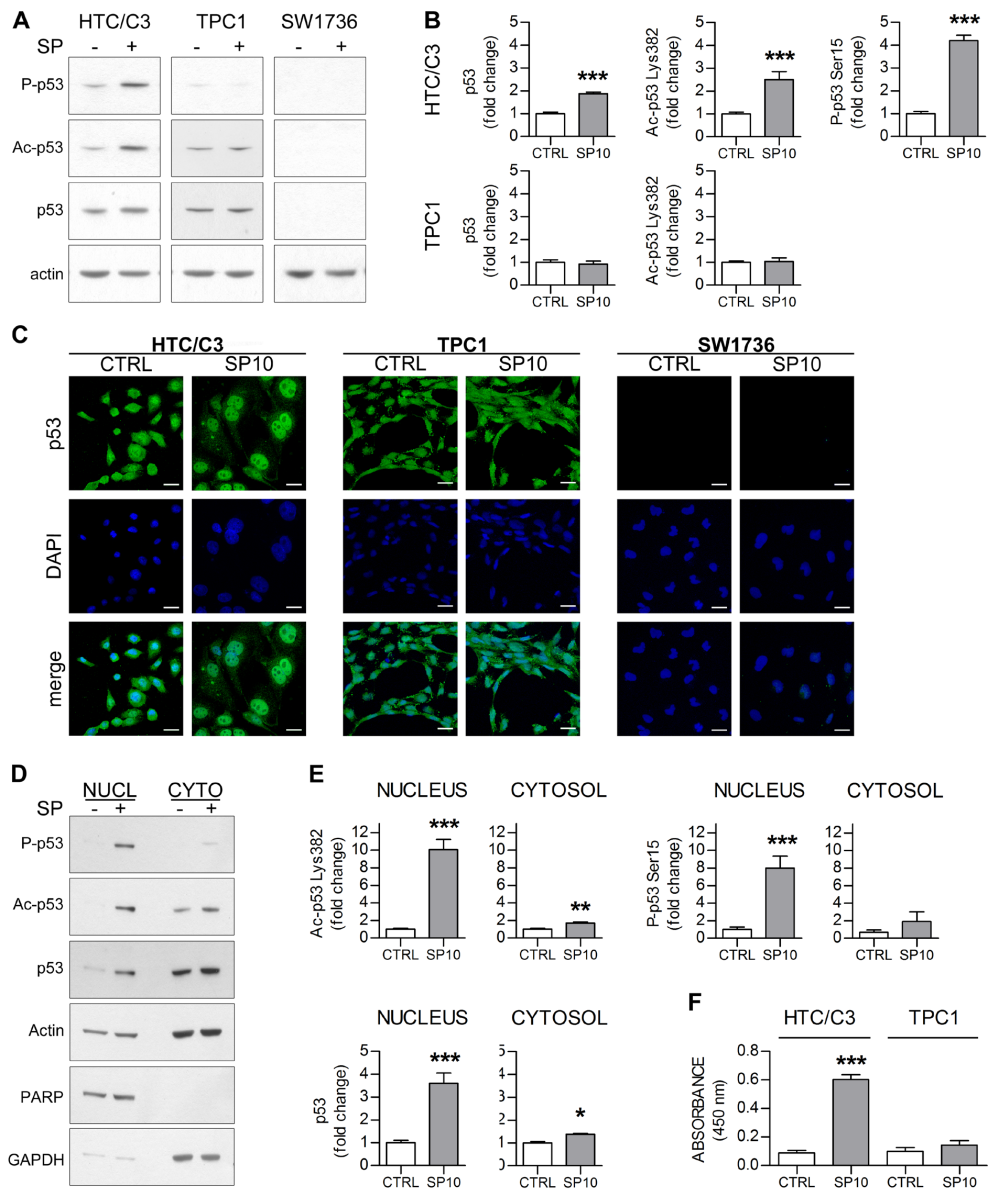
SP induces p53 nuclear translocation and activation in HTC/C3 cells HTC/C3, TPC1 and SW1736 cells were incubated with 10 μM SP (SP10) or equivalent amount of DMSO (CTRL) for 96 hours; p53 nuclear translocation, its activating post-translational modifications and DNA binding capacity was examined. **A.** Representative images of whole cell lysates western blots showing the levels of Ser 15 phosphorylated (P-p53), Lys 382 acetylated (Ac-p53) and total p53; actin was used as loading control. **B.** Densitometric analysis of whole cell lysates western blots. **C.** Confocal microscopy images showing changes in p53 (green staining) subcellular localization following SP treatment. DAPI (blue staining) was used as nuclear marker. Scalebars: 30 μm. **D.** Representative images of western blots of the nuclear (NUCL) and cytosolic (CYTO) extracts of HTC/C3 cells. The amount of Ser 15 phosphorylated (P-p53), Lys 382 acetylated (Ac-p53) and total p53 was examined. Actin was used as loading control, PARP was used as nuclear marker and GAPDH was used as cytoplasmic marker. **E.** Densitometric analysis of the western blots of the HTC/C3 subcellular fractions. **F.** SP-induced changes in the DNA binding capacity of p53 in the two model cell lines expressing p53 protein.. *p < 0.05, **p < 0.01, ***p < 0.001 *vs*. control.

The investigation of p53 levels and post-translational modification showed that 10 μM SP treatment leads to p53 phosphorylation at Serine 15 and acetylation at Lysine 382 only in HTC/C3 cells whereas no significant modifications were detected in TPC1 cells; a significant increase in p53 levels, compatible with p53 activation and stabilization, was detected in HTC/C3 cells (Figure 2A and 2B), as these modification deeply affect p53 half-life and activity [28]; the fact that after SP treatment they were induced only in the p53 mutated cell lines made this pathway noteworthy of further investigations.

One of the main mechanisms of mutant p53 inactivation is the retention in cytoplasm, as most of p53 post-translational modifications take place in the nucleus [28]. Immunofluorescence experiments showed that 10 μM SP treatment leads to p53 nuclear translocation only in HTC/C3 cells whereas no significant translocation could be detected in TPC1 cells (Figure 2C). In HTC/C3 cells these results were confirmed by cellular fractioning experiments, showing a significant increase in the p53 nuclear fraction (Figure 2D). Moreover, a significant increase in p53 Serine 15 phosphorylation and Lysine 382 acetylation was found only in the nuclear fraction of HTC/C3 cells (Figure 2D and 2E). In accordance with the detected post-translational modifications, an increase in p53 DNA binding activity was detected in HTC/C3 cells after SP treatment, whereas no similar changes were observed in TPC1 cells (Figure 2F).

These results suggested that not only SP treatment is able to induce nuclear translocation of the mutant p53, but also promotes its functional activation. Therefore, the effects of SP treatment on the p53-downstream effector p21 were investigated either without or in the presence of Ischemin. This is a synthetic suppressor of p53 transcriptional activity through the inhibition of its binding to the cofactor CBP after Lysine 382 acetylation [29].

**Figure 3:**
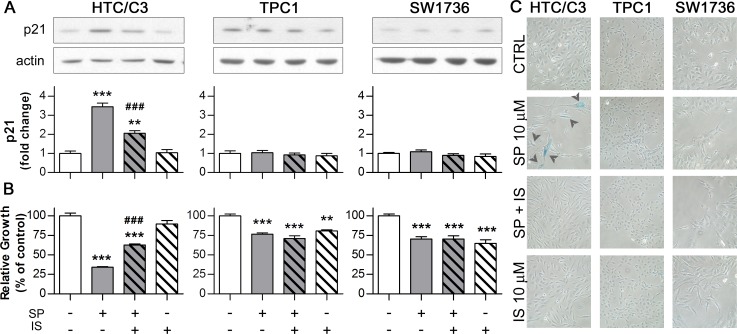
activated p53 induces senescence through its effector p21 Cells were incubated with 10 μM SP either in the presence of or without p53 inhibitor Ischemin (IS, 10 μM, subtoxical concentration determined in TPC1 cells) for 96 hours followed by the assessment of p21 expression, growth inhibition, morphological changes and senescence induction. Corresponding amounts of DMSO (CTRL) or Ischemin (IS) alone were used as controls. In the panel A and B, the p21 expression and the growth inhibition data are aligned with the respective treatment scheme below the bar diagram of growth inhibition. **A.** Representative images of western blots and their quantification illustrating the effect of SP and Ischemin treatment on p21 expression; actin was used as loading control **B.** Differential effects of Ischemin on the SP-induced growth inhibition in HTC/C3, TPC1 and SW1736 cells. **C.** SP-induced morphological changes and senescence (detected as blue senescence-specific X-gal staining - arrowheads) are reverted by Ischemin treatment in HTC/C3 cells. **p < 0.01, ***p < 0.001 *vs*. control; ###*p* < 0.001 *vs*. SP treated cells.

We found that exposure to SP leads to a significant increase in p21 level only in HTC/C3 cells. This increase is, at least partially, dependent on p53 activity, as pre-incubation with Ischemin significantly reverted p21 induction (Figure 3A). Moreover, inhibition of p53 activity partially reduced the anti-proliferative effect of SP on HTC/C3 cells (Figure 3B), supporting the involvement of p53/p21 pathway modulation in SP mechanism of action.

In order to investigate the unknown mechanisms of p21 induction after SP treatment [11, 14, 30], 43 kinases and 2 related total proteins were simultaneously analyzed with Human Phospho-Kinase Antibody Array in HTC/C3 cells. Significant changes in the level of β-catenin and other proteins involved in cell migration and growth control were detected after 60 hours of incubation with 10 μM SP (Supplementary Figure 1, Supplementary Table 2). After 96 hours of treatment, the only modifications conserving the level of significance were p53 phosphorylation at Serine 15, FAK phosphorylation at Tyrosine 397 and β-catenin increases (Supplementary Figure 1, Supplementary Table 2).

One of the main consequences of p53/p21 pathway activation is the induction of cellular senescence. In fact *TP53* mutant cells surviving 96 hours of SP treatment appeared flattened, bigger, with increased amount of cytoplasmic vacuoles and dishomogeneous content. Up to 20% of the surviving HTC/C3 cells showed the senescence-specific X-gal staining. These effects were abolished by inhibition of p53 transcriptional activity through Ischemin pre-treatment (Figure 3C).

The presence of nuclear abnormalities such as heterochromatic foci, multinucleation and nuclear blebbing, lysosomal compartment expansion and positive γ-H2A.X staining in a large fraction of SP treated HTC/C3 cells (Supplementary Figure 2) confirmed the presence of cellular senescence [31-32]. To date, increases in cell dimensions and in p21 expression following SP treatment have been occasionally reported [27, 33]. On the other hand, SP-induced p53-dependent premature senescence has never been reported. These data support that mutant p53 reactivation and senescence induction may contribute to the overall antiproliferative effect of SP.

### SP induces cell death through mitotic catastrophe

The activation of p53/p21 pathway is responsible for SP cytostatic action by inducing premature senescence in cells which survived SP treatment; however the exact mechanism by which low doses of SP exert cytotoxic actions remains elusive. It has been reported previously that SP inhibits distinct kinases involved in cell cycle regulation and endoreduplication [11-12, 34]. We investigated whether SP is able to induce mitotic alterations in our cell lines.

**Figure 4:**
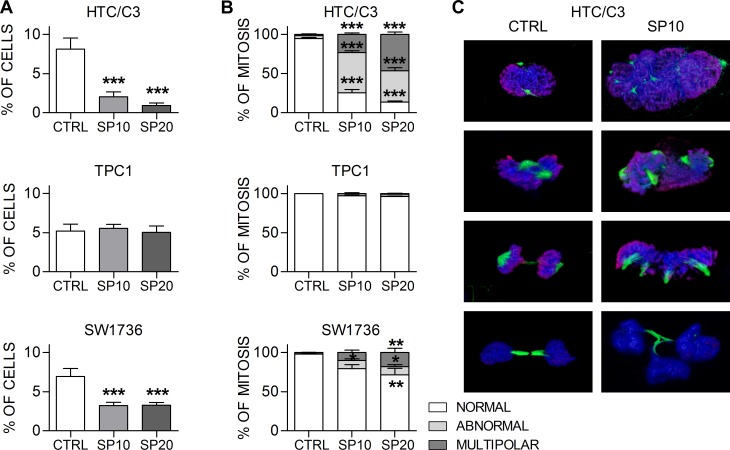
SP induces mitotic alterations in sensible cells Cells were incubated with 10 μM (SP10) and 20 μM (SP20) SP or equal amount of DMSO (CTRL) for 72 hours and mitosis were analyzed with confocal microscopy. **A.** percentages of mitotic cells per microscopy field. **B.** percentages of normal, abnormal and multipolar mitosis. **C.** Representative 3D reconstructions from confocal images of normal mitotic figures in DMSO treated (CTRL) and abnormal mitotic figures in SP treated HTC/C3 cells. Staining shows Ser 10 Phospho-Histone H3 (red) and mitotic spindle (tubulin, green); DNA was labeled with DAPI (blue). *p < 0.05, **p < 0.01, ***p < 0.001 *vs*. control.

Our results show that SP significantly affects mitosis in HTC/C3 cells and, to a lesser extent, in SW1736 cells, starting from 48 hours of incubation. In addition to the reduction in the frequency of mitotic cells (Figure 4A), morphological analysis of mitotic figures revealed an increase in the percentage of abnormal and multipolar mitotic forms (Figure 4B and 4C), defects that usually lead to mitotic slippage and aberrant division. No similar alterations were detected in TPC1 cells. Considering that aberrant mitosis and unbalanced DNA content can lead to death through mitotic catastrophe or to premature senescence, we hypothesize that mitotic cell death is the main anti-proliferative mechanism of SP, while induction of premature senescence in cells surviving the cytotoxic action is a later event and a crucial additional property that starts configuring SP as a pleiotropic anticancer agent.

### SP effects are the result of alterations in microtubules network

**Figure 5:**
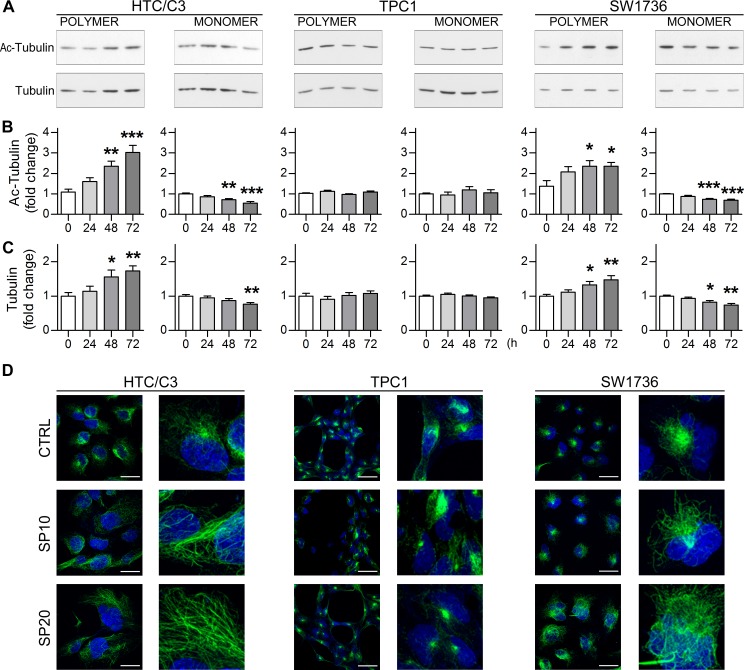
SP alters microtubule architecture in a dose-dependent manner The effect of SP on tubulin polymerization and acetylation was analyzed. **A.** Representative images of western blots of polymeric and monomeric tubulin fractions and their acetylation levels at Lys 40 following treatment with 10 μM SP up to 72 hours. **B.** Quantification of polymeric and monomeric tubulin acetylation levels. **C.** Quantification of the relative amounts of tubulin fractions. **D.** Confocal images of whole cell acquisitions (left columns) and relative 4X magnification details (right columns) showing acetylated microtubules architecture after DMSO (CTRL), 10 μM (SP10) and 20 μM (SP20) SP incubation for 72 hours. Acetylated microtubule network, green staining; nuclei labeled with DAPI, blue staining; scalebars: 30 μm. *p < 0.05, **p < 0.01, ***p < 0.001 *vs* day 0.

High concentrations SP were shown to induce tubulin polymerization *in vitro* [33]. Microtubule poisons are well known anticancer drugs causing primary failure in mitosis progression and mitotic cell death with a stronger effect in p53 deficient cells [35-37]. Hence the effect of SP on the microtubule network organization of the cells was examined. A semi-quantitative tubulin fractionation assay revealed that SP induces a significant increase in tubulin polymerization in p53-mutant and pseudo-null cells (Figure 5A and 5C) together with a consistent increase in its acetylation at Lysine 40 (Figure 5A and 5B). This modification is usually found on stable microtubules but can result in mitosis block or abnormal chromosome segregation, when present at supraphysiological levels. Confocal microscopy revealed a complete upheaval of normal microtubule architecture in HTC/C3 cells. While the typical polarized acetylated fibers emerging from the microtubule-organizing center (MTOC) were clearly recognizable in untreated cells, the microtubules appeared thicker, bundle-like, widespread through the cytosol without an organizing center and completely unrelated to the nucleus after SP treatment. Importantly, only minor alterations were detected in TPC1 cells while an intermediate effect was detected in SW1736 cells (Figure 5D) Therefore, alterations in tubulin dynamics may represent one of the main effects of SP, and elucidation of the exact mechanisms by which SP induces microtubule alterations represents a key point for its further development as anticancer drug.

### SP exerts its main effects through direct inhibition of ROCK/HDAC6 pathway

In a previous screening, SP significantly inhibited the activity of ROCK [38], a kinase recently described to control tubulin and β-catenin stability through their main deacetylating enzyme HDAC6 [39-41]. Importantly, both enzymes are involved in cancer invasiveness and metastasization, and their activity was found to be increased in several undifferentiated cancers [24-25, 42-45].

**Figure 6:**
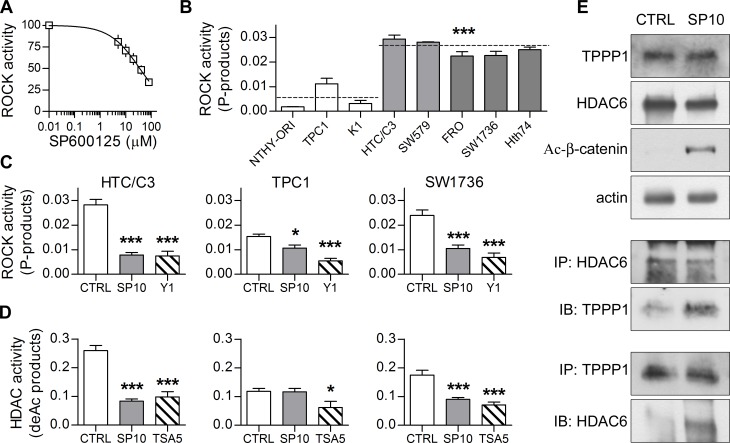
SP acts through ROCK/HDAC6 pathway inhibition Basal status of ROCK/HDAC6 pathway and SP-induced modifications were analyzed. **A.** Cell-free enzyme activity assay shows that SP directly inhibits ROCK in a dose-dependent manner. **B.** Quantification of basal ROCK activity in our thyroid cell lines. Statistical significance is shown for undifferentiated versus differentiated thyroid cancer cell mean values. **C.** Quantification of ROCK activity in HTC/C3, TPC1 and SW1736 cells after incubation with 10 μM SP (SP10) or equivalent amount of DMSO (CTRL) for 72 hours; 1 μM ROCK inhibitor Y27632 (Y1) was used as control. **D.** Quantification of cytoplasmic HDAC activity in HTC/C3, TPC1 and SW1736 cells after incubation with 10 μM SP (SP10) or equivalent amount of DMSO (CTRL) for 72 hours; 5 μM HDAC inhibitor Trichostatin A (TSA5) was used as control. **E.** Representative images of western blots showing total amount of HDAC6, TPPP1 and Lys 49 acetylated β-catenin (upper blots) and co-immunoprecipitation experiments (central and lower blots) after incubation of HTC/C3 cells with 10 μM SP (SP10) or equivalent amount of DMSO (CTRL) for 72 hours; actin was used as loading control. IP immunoprecipitated protein, IB immunoblot. *p < 0.05, ***p < 0.001.

Therefore, we first confirmed the dose dependent inhibitory effect of SP on the ROCK activity in a cell-free enzyme inhibition assay (Figure 6A). Subsequently, basal ROCK activity was examined, showing that cell lines derived from undifferentiated thyroid cancers have a significantly higher activity than those derived from thyrocytes, a tendency accurately reflected by our model cell lines (Figure 6B). Noticeably, in HTC/C3 and SW1736 cells 10μM SP could counteract and reduce the ROCK activity to the level of WDTCs, whereas the inhibition was considerably lower in TPC1 cells (Figure 6C).

As a second step, we examined whether these results were reflected by the downstream pathway. Indeed, variations in cytoplasmic HDACs activity reflected the ROCK ones. The basal cytoplasmic HDACs activity in HTC/C3 and SW1736 was higher than in TPC1, and SP treatment exerted a significant inhibitory effect (Figure 6D).

In particular, ROCK regulates the activity of HDAC6 through direct phosphorylation of TPPP1. TPPP1/HDAC6 interaction results in the inhibition of HDAC6 activity while TPPP1 phosphorylation causes HDAC6 release and activation. HDAC6 then regulates microtubule and β-catenin stability [40].

Co-immunoprecipitation experiments showed that SP treatment significantly increases the interactions between TPPP1 and HDAC6 (Figure 6E), a modification accompanied by a significant enhancement of the acetylation of β-catenin (Figure 6E) and tubulin (previously reported in Figure 5A, 5B and 5D).

In addition, further important consequences of the ROCK/HDAC6 pathway inhibition include the impairment of cell migration and invasiveness [39-40, 46-47]. Therefore we examine if SP treatment influences these processes.

**Figure 7:**
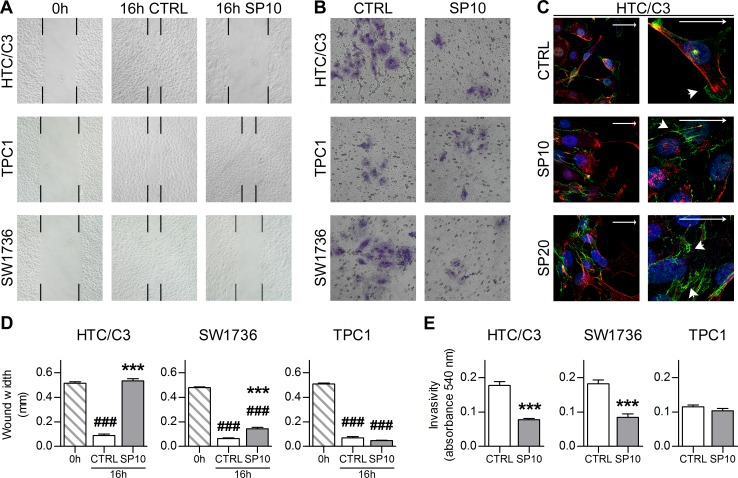
SP inhibits cellular migration and invasiveness **A.** Representative images of wound-healing assays with 10 μM SP (SP10) or equivalent amount of DMSO (CTRL) treatment. Black marks indicate wound edges at 0 and 16 hours post wound. **B.** Representative images of crystal violet stained cells from invasion assays after 10 μM SP (SP10) or equivalent amount of DMSO (CTRL) treatment. **C.** Effects of 10 μM SP (SP10), 20 μM SP (SP20) or equivalent amount of DMSO (CTRL) treatment on β-catenin localization in migrating HTC/C3 cells and its details at higher magnification. Scalebars represent 30 μm throughout the figure and indicate free edge direction. Arrowheads indicate β-catenin staining at cell membrane leading edge (CTRL) and intercellular junction (SP treated). Staining shows β-catenin (green), nucleus (DAPI, blue) and cell membrane (WGA, red). **D.** Quantification of wound width in wound-healing assays. **E.** Quantification of invasivity by lower-chamber cells solubilization and absorbance reading. ###*p* < 0.001 *vs*. 0 hours post wound, ***p < 0.001 *vs*. CTRL.

A Wound-Healing assay revealed that SP is able to actively inhibit undifferentiated cancer cell migration. After SP treatment, TPC1 cells replenished the scratch whereas no progression toward the free edge was achieved by HTC/C3 cells and only partial replenishment was seen in SW1736 cells at 16 hours post wound (Figure 7A and 7D). In agreement with these data, an invasion assay showed a significant inhibition by SP of cell invasivity potential in HTC/C3 and SW1736, while TPC1 showed a lower but unaltered invasiveness (Figure 7B and 7E).

As last step, we investigated β-catenin localization because it can act as pro-proliferative factor when it is cleaved from the cell membrane and relocated to the nucleus. In both resting and migrating HTC/C3 cells, β-catenin is localized at the cell membrane (Figure 7C, arrowheads). Moreover, SP treatment enhanced β-catenin signal and inhibited its relocation from the intercellular adhesion surface toward the leading edge of cell migration as indicated by arrowheads (Figure 7C). Noteworthy, in none of the cited conditions we observed an increased nuclear staining for β-catenin.

### ROCK pathway in human thyroid cancer and correlation with p53 activity

Since no data about ROCK involvement in thyroid cancer development are currently available, we collected 12 tissue samples from patients that underwent thyroidectomy for compressive multinodular goiter, PTC or PDTC (Table 1). Genetic screening revealed that patient 9 is heterozygous for *TP53* c.920-2A > G, a variant causing alternative splicing of exon 9 and premature stop codon, while patients 5, 6, 8 and 10 resulted heterozygous for BRAF^V600E^ variant, and patient 8 carried C228T TERT promoter mutation.

**Figure 8:**
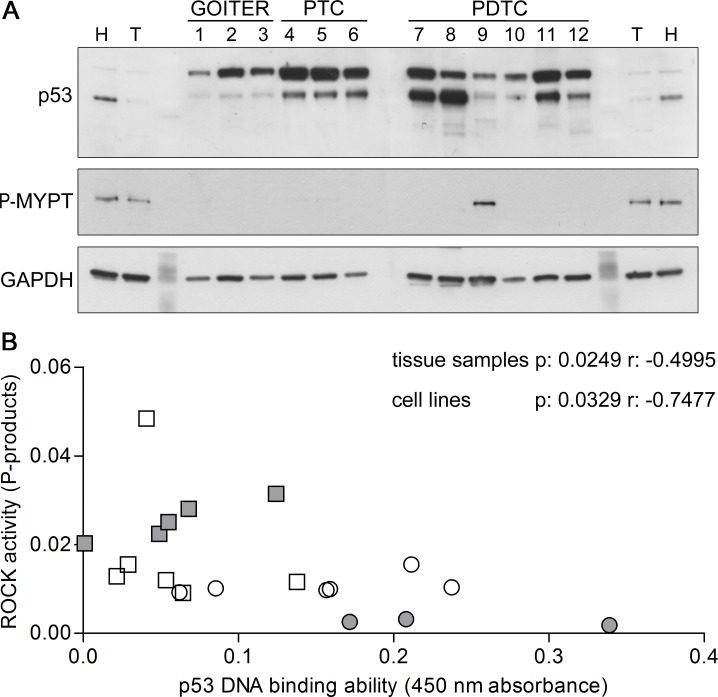
p53 and ROCK activity in thyroid tissue samples **A.** Representative images of western blots of thyroid tissue samples derived from multinodular goiters (GOITER), papillary thyroid cancers (PTC) and poorly differentiated thyroid cancers (PDTC) showing the relative amount of p53 and Thr 696 phosphorylated MYPT1 (P-MYPT); GAPDH was used as loading control. An aspecific band of approximately 70 kDa was detected by p53 antibody. **B.** p53 DNA binding ability plotted versus ROCK activity graph of thyroid tissue samples (white symbols) and cell lines (grey symbols) showing significant negative correlation. Differentiated samples are reported as circles while poorly differentiated and anaplastic samples are reported as squares.

Western blot analysis of protein extracts showed variable alterations in p53 levels in all cancer samples, with significant increases at densitometry in PDTCs ones compared to goiters ones (7.671±1.198 *vs*. 1.989±0.2921, respectively, *p* < 0.01). Low molecular weight bands of approximately 40-46 kDa were detected only in PDTCs samples (Figure 8A). These bands likely represent truncated forms of p53 that are known to be overexpressed in a significant fraction of undifferentiated cancer cells [48-49].

Interestingly, the highest phosphorylation levels of a direct ROCK substrate, MYPT, were detected in the patient harbouring the p53 mutation.

In addition, the plot of p53 DNA binding ability versus ROCK activity showed a strong negative correlation in thyroid tissue samples as well as in cell lines with a p-value of 0.0249 and 0.0329, respectively and Pearson's r of −0.4995 and −0.7477, respectively (Figure 8B) providing a suggestive coupling between the two main SP effects.

**Table 1:** Genetic and clinical features of the human tumors samples

Patients	Histological data	Genetic analyses			Relevant clinical data
		*BRAF*	*TP53*	*TERT*	
4	Papillary, pT3N1b	WT	WT	nd	WDTC, persistent disease, lung metasstasis
5	Papillary, pT1Nx	p.V600E	WT	nd	WDTC, cured
6	Papillary, pT3N0	p.V600E	WT	WT	WDTC, cured
7	PDTC, pT4aN1	WT	WT	nd	External radiotherapy, surgery for endotracheal recurrence, persistent disease
8	PDTC, pT3N1	p.V600E	WT	p.C228T	Biochemical persistence
9	PDTC, pT3N1b	WT	c.920-2A>G	WT	Resistant to tyrosine-kinase inhibitor, disease-related death at 26 years
10	PDTC, pT4aN1b	p.V600E	WT	WT	Persistent disease, local recurrence
11	PDTC, pT4aNx	WT	WT	WT	Persistent disease, lost at follow-up
12	PDTC, pT4aN1b	WT	WT	nd	Local recurrence, disease-related death at 77 years

The table summarize the histological, genetic and clinical data of patients with thyroid malignancies.

WDTC, well differentiated thyroid cancer; PDTC, poorly differentiated thyroid cancer; nd, not done.

## DISCUSSION

In the present study we provide preliminary data on ROCK pathway alterations in thyroid cancer and we prove that SP retains an intriguing pleiotropic potential as anticancer agent.

SP appears highly effective against ATCs and PDTCs *in vitro*, two histotypes of highly lethal malignancies characterized by local invasion, regional and distant metastases, disease recurrence and refractoriness to currently available anticancer therapies, including conventional and new-generation compounds [1, 4]. Importantly, we show that SP is highly effective in blocking thyroid cancer cell growth and migration and in the induction of mitotic catastrophe through direct inhibition of ROCK, a kinase involved in the regulation of cell migration, microtubule dynamics and β-catenin turnover [39-40, 46, 50]. This mechanism of action may be particularly important in anticancer therapy considering that Rho/ROCK pathway is hyperactivated in different human neoplasia and its activity correlates with metastatic disease [24-25, 42-43, 51-52]. No data on Rho/ROCK activation in thyroid cancer were available until now as the only previous study claiming a possible involvement of this pathway was performed on ARO cells [53], which are now known not to be of thyroid origin [54]. Here we show for the first time that ROCK pathway is hyperactivated in PDTCs with defective p53 activity. In fact those derived from PDTCs had the highest levels of ROCK activity, which was reduced to the level of WDTCs upon SP treatment.

**Figure 9:**
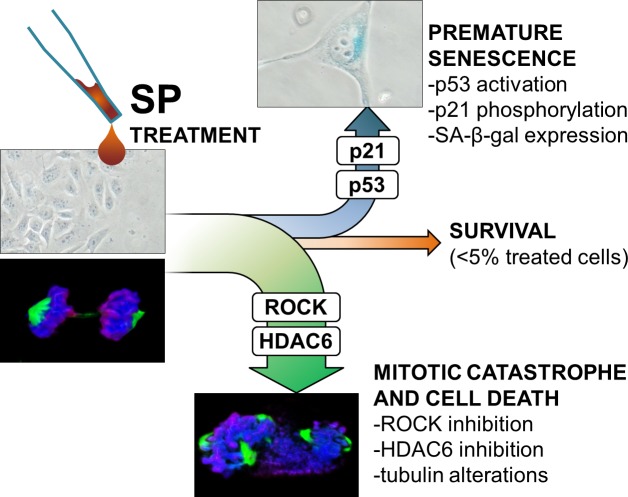
Proposed schematic representation of SP action The scheme shows SP effects on sensitive cancer cells and supposed mechanisms of action as derived from the integration between experimental data and literature background. The alteration of ROCK/HDAC6 pathways leads to tubulin abnormalities and this could be responsible of mitotic catastrophe in the major part of treated cells (∼80 %). Longer incubation with SP leads to induction of premature senescence through p53/p21 pathway activation in a significant amount of surviving cells (∼15 %).

In accordance with a previous work [39], inhibition of ROCK affected HDAC6 function. Here we show that this is mediated by an enhanced TPPP1-HDAC6 interactions. These alterations can account for the observed tubulin hyperstabilization and consequent mitotic abnormalities. The inability to proceed through the mitotic process is one of the known possible causes of cell death, mainly through mitotic catastrophe induction, and possibly accounts for the largest part of the anti-oncogenic effects of SP here described (Figure 9). Since an increasing literature background [24, 26, 39] report a strict relation between the inhibition of ROCK/TPP1/HDAC6 pathway and cell motility alterations, it is likely that this inhibition can account for the inhibition of cell migration and invasion observed after SP treatment.

In addition, we provide new insights about the relationship between p53 inactivation and ROCK hyperactivation in thyroid cancer. In fact the highest ROCK activity was detected in the only PDTC with *TP53* mutation. Interestingly, this tumor was particulartly aggressive (Table 1). Our results demonstrate a strong inverse correlation between p53 DNA binding ability and ROCK activity in both tissue samples and cell lines. These findings are in agreement with previous reports of RhoA/ROCK pathway hyperactivation and increased invasiveness in cells with loss of p53 activity [18, 20]. Thus, the SP strong inhibitory effect against ROCK can be considered as the sum of direct inhibition through kinase binding and indirect inhibition through p53 pathway restoration. This perspective can also explain the differences observed among p53 mutated and p53 null ones, as in the latter the second mechanism of action is not possible.

We also show here that premature senescence is induced by SP in the HTC/C3 cells harboring *TP53* inactivating missense mutations. Since premature senescence appears at later time points than mitotic catastrophe and affect SP survived cells, one hypothesis can be that it is a secondary mechanism developed by cells surviving the SP-induced polyploidy and cell death (Figure 9). It is conceivable that the nodal point of this event is its dependence on the reactivation of the expressed p53 variants. The reactivation process restores cell responsiveness to chromosomal aberrations induced by microtubule alterations and results in senescence induction. On the other hand, the reactivation of p53 activity can trigger the so-called Oncogene-Induced Senescence (OIS). It has been demonstrated that the constant MAPK intracellular signaling induced by RAS or RAF mutations causes OIS rather than malignant transformation when not accompanied by other fundamental alterations that disrupt cell cycle control. The restoration of p53 activity can thus inhibit cell proliferation by allowing the activation of OIS [32]. Interestingly, these processes have been demonstrated also in thyroid cancer models, mainly in relation to the presence of BRAF^V600E^ alteration, one of the main target of new TKI anticancer therapies [7, 55-57]. The speculation about OIS induction after p53 activity restoration by SP is supported by the selectivity against cell lines harbouring p53 mutations and the lack of effects on wild type or p53 pseudo-null ones.

Although the exact mechanism by which SP influences p53 activity still needs to be clarified, the differences in the growth inhibition may be partially dependent on the greater ROCK inhibition observed in HTC/C3 cells versus the p53 pseudo-null ones. In fact, as ROCK activity is enhanced by p53 alterations [18, 20], the restoration of p53 activity can negatively influence the ROCK one. The induction of senescence in HTC/C3 surviving clones in contrast to the maintenance of the proliferation phenotype in SW1736 ones then adds further difference to the final growth inhibitory result of SP treatment.

In conclusion, our studies brought new evidence of ROCK/HDAC6 pathway involvement in thyroid tumors aggressiveness.

By targeting this pathway and the well known p53 alterations, SP inhibits cell replication and migration at the same time, the two major processes involved in cancer development and dissemination thus revealing novel anticancer effects of SP in highly aggressive thyroid cancer cells. These effects may render SP a potential therapeutic agent in undifferentiated malignancies.

## MATERIALS AND METHODS

### Chemicals

Cell culture reagents were purchased from Life Technologies. SP600125 (1,9-Pyrazoloanthrone), MTT (3-(4,5-Dimethyl-2-thiazolyl)-2,5-diphenyl-2-tetrazolium-bromide), Monoclonal anti-β-Catenin antibody 6F9, Monoclonal Anti-α-Tubulin clone DM1A, Monoclonal Anti-Acetylated Tubulin Clone 6-11B-1 were purchased from Sigma-Aldrich. p53 antibody, Phospho-p53 (Ser46) antibody, p53 (1C12) Mouse mAb, p21 Waf1/Cip1 (DCS60) Mouse mAb, p21 Waf1/Cip1 (12D1) Rabbit mAb, Acetyl-p53 (Lys382) antibody, Phospho-p53 (Ser15), antibody, Phospho-Histone H2A.X 20E3 antibody, HDAC6 D2E5 antibody and Acetyl- β-Catenin (Lys49) antibody were purchased from Cell Signaling Technologies. ProLong Gold Antifade Reagent with DAPI, Wheat Germ Agglutinin Alexa-Fluor 594 Conjugate, Alexa-fluor conjugated and HRP conjugated secondary antibodies were purchased from Life Technologies. Purified Mouse Anti-Actin Ab-5 was purchased from BD Biosciences. Ischemin (MS120) and P-MYPT antibody were purchased from Merk Millipore. Phospho-Histone H3 pSer10 Antibody, TPPP/p25 antibody, TPPP antibody, Restore Western Blot Stripping reagent were purchased from Thermo Scientific. Protein G sepharose was purchased from GE Healthcare Life Sciences.

### Cell culture

Cell lines used as model of normal thyrocytes (NTHY-ORI 3-1), well differentiated thyroid cancer (TPC1, K1), poorly differentiated thyroid cancer (HTC/C3 and SW579) and anaplastic thyroid cancer (FRO, SW1736 and Hth74) were a kind gift of Dr. Italia Bongarzone (Milan, Italy). Cells were grown as monolayers in 100 mm plastic culture dishes with appropriate medium supplemented with penicillin and streptomycin and kept in a humidified incubator at 37°C under 5 % CO2. Specific cell lines origins and pathogenetic alterations are reported as part of supplementary material and methods. All experiments were performed with cell lines in between 7th and 13th passage. All cell lines were routinely screened for mycoplasma contamination with MycoAlert Mycoplasma Detection Kit (Lonza).

### Proliferation assay

Cell proliferation was evaluated utilizing the 3-(4,5-dimetylthiazole-2-yl)-2,5-diphenyltetrazolium bromide (MTT) assay as described before [58]. On the day of the assay, MTT solution was added to each well to a final concentration of 0.5 mg/ml 3 hours before measurement. Formazan crystals were then solubilized in 200μL of EtOH:DMSO 1:1 solution. Absorbance was red at 540 nm using ELx800 Absorbance Microplate Reader (BioTek).

### Thyroid tissue samples

Thyroid tissues from benign goiters and malignant tumors were collected during surgery, immediately frozen and stored at −80°C. All the patients gave informed consent for genetic analysis and tissue studies. All specimens were reviewed by a trained pathologist to confirm the diagnosis. Goiters were used as reference control. Tumors were classified and staged according to the 7th edition of the TNM staging (Table 1),

### DNA extraction and sequencing

Around 10 mg of tissue was cut from frozen samples. DNA Extraction was performed on cut samples with Wizard Genomic DNA Purification Kit (Promega) following manufacturer's instruction.

Bidirectional Sanger sequencing of the genomic DNA from patients’ samples was performed in order to reveal potential mutations in the coding exons of the *TP53* gene, of *TERT* and of exon 15 of the *BRAF* gene spanning the mutation hotspot at codon 600. Primers and detailed PCR protocol are reported in supplementary material and methods. PCR products were cleaned up with ExoSAP-IT (Affymetrix) according to the manufacturer's protocol. Dye terminator cycle sequencing was performed with BigDye Terminator v3.1 Cycle Sequencing Kit (Life Technologies) according to the manufacturer's instructions and, following gel filtration cleanup of the reaction products, sequencing was performed on Applied Biosystems 3500 Genetic Analyzer (Life Technologies).

### Protein extraction and western blot analysis

Western Blot experiments were performed as previously reported [59]. For detection of Phospho-proteins cells were lysed with SDS sample buffer (62.5 mM Tris-HCl pH 6.8, 2% SDS) supplemented with protease and phosphatase inhibitors, immediately heated for 3 min at 95°C and sonicated.

Cellular fractioning experiments were performed with NE-PER Nuclear and Cytoplasmic Extraction Reagents (Thermo Scientific) kit following the manufacturer's instruction.

Around 10 mg of tissue samples were homogenized and lysed in RIPA buffer (10 mM Tris-HCl pH 7.5, 500 mM NaCl, 0.1% SDS, 1% NP40, 1% Na-deoxycholate, 2 mM EDTA) supplemented with protease inhibitors. After incubation on rocking platform for 30 minutes at 4°, samples were centrifuged at 15000g for 20 minutes at 4°C and supernatant was collected to new tubes.

All samples were quantified and immediately stored at −80.

Protein extracts were separated on NuPage 4-12% Bis-Tris Gels (Life Technologies) and transferred with iBlot System (Life technologies). Co-Immunoprecipitation samples were separated on 4-20% Mini-PROTEAN TGX Precast Protein Gels (Biorad) and transferred with iBlot System (Life technologies)

Membranes were blocked with 5% milk TBS-T, probed overnight at 4°C with the indicated primary antibody and incubated with the appropriate HRP-conjugated secondary antibody for 1 h at room temperature. Detection was performed using the ECL-plus kit. Bands of interest were quantified using ImageJ software version 1.47.

### p53 DNA binding assay

The assay was performed with p53 transcriptional factor assay kit (Cayman Chemicals) following manufacteur's instruction on nuclear extracts obtained with NE-PER Nuclear and Cytoplasmic Extraction Reagents. Colorimetric reaction was red at 450nm with ELx800 Absorbance Microplate Reader (BioTek).

### Human phospho-kinase antibody array

43 kinases and 2 related total proteins were analyzed with Human Phospho-Kinase Antibody Array (R&D Systems) following manufacturer's instructions.

### Senescence assay

Senescence induction was assayed with Senescence Cells Histochemical Staining Kit (Sigma-Aldrich) following the manufacturer's instruction. Six fields per well were acquired with Kodak Camera mounted on Olympus CK2 microscope with A10PL 10X objective.

### Tubulin polymerization assay

Tubulin monomer (soluble) and polymer (insoluble) forms were separated by centrifugation in hypotonic buffer and relative amounts were analyzed by electrophoresis and Western blotting as previously described [60]. Briefly cells were washed twice with warm PBS and then lysed with 100μl of hypotonic buffer (1 mM MgCl_2,_ 2 mM EGTA, 0.5% NP40, 20 mM Tris-HCl pH 6.8) plus freshly added 2 mM PMSF and protease inhibitor cocktail for 10 minutes at 37°C. Cells were collected in tubes and the particulate fraction was separated from the soluble cytosolic fraction by centrifugation at 14000g for 10 minutes. Surnatant containing the monomeric tubulin was immediately collected and transferred to a new tube while the pellet containing the polymeric form was resuspended in equal amount of hypotonic buffer. All samples were sonicated and analyzed by electrophoresis and western blotting as described above.

### Immunofluorescence and confocal microscopy

For cell membrane visualization, living cells were incubated with 5 μg/ml Wheat Germ Agglutinin Alexa-Fluor 594 Conjugate (Life Technologies) in PBS for 10 minute at 37°C in 5% CO2 prior to samples fixation. Samples were washed three times with pre-warmed PBS and fixated by incubation for in pre-warmed 2% PFA in PBS 10 minutes. After washing with PBS cells were pearmeabilized with 0.2% Triton-X in PBS for 10 minutes and then blocked with 5% goat serum PBS at room temperature for 1 hour. Samples were incubated over night at 4°C with primary antibody solution. On the following day cells were washed three times in PBS, and 1 hour incubation was performed with appropriated secondary antibody solution. Samples were mounted on microscope slides with 15 μl of ProLong Gold Antifade Reagent with DAPI (Life Technologies).

Images were acquired with Nikon EclipseTi-E inverted microscope with IMA10X Argon-ion laser System by Melles Griot; all images were acquired with CFI Plan Apo VC 60X Oil (Nikon) objective except those for mitotic index determination which were acquired with CFI Plan Apo VC 20X (Nikon) objective. For p53 imaging single acquisition was performed on nuclear plane. For H2A.X foci whole nuclei were acquired with Z-series acquisition, 0.15 μm steps. For microtubules and migration imaging whole cells were acquired with Z-series acquisition, 0.15 μm steps; for mitosis whole cells were acquired with Z-series acquisition, 0.1 μm steps and 3D structure reconstucted with NIS-Elements AR software. Mitotic figures were classified following Gisselsson description [61] after staining with DAPI, anti-tubulin and anti-P-Histone H3 antibodies.

### Wound healing assay

Confluent cells were scraped with a p200 tip, washed with PBS and returned to control or SP supplemented medium. Images were acquired at 0 and 16h post-wound with Kodak EasyShare C195 camera on Olympus CK2 microscope with A10PL 10X objective. Wound dimensions were quantified with ImageJ software version 1.47.

### Invasion assay

Cell invasivity was assayed with CytoSelect 24-Well Cell Invasion Assay (Cell Biolabs, Inc) following the manufacturer's instruction with minor modifications previously described [62]. 5 individual fields per insert were acquired with Kodak EasyShare C195 camera on Olympus CK2 microscope with A20PL 20X objective. After imaging, crystal violet stained cells were solubilized and absorbance was red at 540 nm using ELx800 Absorbance Microplate Reader (BioTek).

### HDAC activity assay

On the day of the assay cell fractioning was performed with NE-PER Nuclear and Cytoplasmic Extraction Reagents. Cytosolic extracts were processed with Epigenase HDAC Activity/Inhibition Direct Assay Kit (Epigentek) following the manufacturer's instructions. Colorimetric reaction was detected at 450nm using ELx800 Absorbance Microplate Reader (BioTek).

### ROCK activity assay

On the day of the assay cells or tissue samples were extracted with specific buffer (50 mM Tris-HCl pH 7.5, 0.1 mM EGTA and 0.1% 2-mercaptoethanol) [63] and ROCK activity measured with Rho-associated Kinase (ROCK) Activity Assay (Millipore) following the manufacturer's instructions. The same kit was used for direct activity inhibition measurement. ROCK kinase was incubated with increasing concentration of SP and constant concentrations of ATP and MgCl_2_. Colorimetric reaction was detected at 450nm using ELx800 Absorbance Microplate Reader (BioTek).

### Co-Immunoprecipitation

Co-Immunoprecipitation was performed as previously described [64], with minimal modifications.

HTC/C3 cells were lysed in buffer containing 50 mM Tris–HCl (pH 7.4), 150mM NaCl, 1% Triton X-100 supplemented with protease inhibitors. 500 μg of lysate was pre-cleared with 10 μL of Protein G sepharose beads for 1 hours at 4°C in agitation. Cleared lysates were incubated with ∼2 μg of appropriated primary antibodies overnight at 4°C with agitation. The following day 100 μl of Protein G sepharose beads were added and samples incubated at 4°C for 2 hours. Protein complexes were precipitated through centrifugation at 15000g for 10 minutes, washed three times and boiled. Samples were centrifugated at 15000g for 10 minutes and surnatant was analyzed by electrophoresis and western blotting as described above.

### Statistical analysis

Values are expressed as means ± SEM. Statistical analysis were performed with GraphPad Prism Software, version 5.04. Student's t-test was performed for two variable comparison while one-way ANOVA followed by Bonferroni's post-hoc test was used to evaluate statistical significance between more than two variables. All experiments were performed at least four times in duplicate, with the exception of Human Phospho-Kinase Antibody Array that was performed once and read in triplicate.

## SUPPLEMENTARY MATERIAL FIGURES, TABLES AND REFERENCES


