# Supplementary material for: SP600125 has a remarkable anticancer potential against undifferentiated thyroid cancer through selective action on ROCK and p53 pathways
Source: Oncotarget. 2015 Sep 22;6(34):36383–99. doi: 10.18632/oncotarget.5799 (PMC4742184; doi:10.18632/oncotarget.5799)
Supplement: Supplementary file 1 [file oncotarget-06-36383-s001.pdf]

SP600125 has a remarkable anticancer potential against undifferentiated thyroid cancer through selective action on ROCK and p53 pathways

Supplementary Material

Cell lines

NTHY-ORI 3-1 cell line was originally obtained from normal human primary thyroid follicular epithelial cells transfected with a plasmid containing an origin-defective large T antigen SV40 genome. They express thyroid epithelial specific features, such as iodide-trapping and thyroglobulin production; Although they acquired growth factor-independency they remain non-tumorigenic in nude mice [1]. NTHY-ORI 3-1 presents diploid status with additional tetraploid clones together with very low frequency of spindle abnormalities and normal centrosomes [2]. They do not present common thyroid carcinoma genetic alterations.

TPC1 cells are derived from a papillary thyroid cancer [3]. TPC1 cells presents diploid status together with an additional minor tetraploid clone with very low frequency of spindle abnormalities and normal centrosomes [2, 4]. They express the thyroid specific transcription factors PAX8, TTF1 and TTF2 [4-5]. They harbour the RET/PTC1 rearrangement, a complex t(1; 10; 21) translocation and a pathogenic *TERT* promoter point mutation as well as a silent polymorphism in *H-RAS* and wild-type status for *PTEN*, *BRAF*, *CTNNB1*, *EGFR*, *K-RAS*, *N-RAS*, *RAF-1*, *PI3K*, *TP53*, and *TRHB* [6-9].

K1 cells were derived from a primary papillary thyroid carcinoma; they share the same genetic profile of the papillary thyroid cancer derived line GLAG-66 [5, 10-11]. They retain thyroglobulin and PAX8 expression, and significant response to TSH stimulation [5, 12]; they are tumorigenic after inoculation in nude mice [12]. presents mainly tetraploid status with with very low frequency of spindle abnormalities and normal centrosomes [2].

They harbour a heterozygous $BRAF^{V600E}$ variant, PI3K p.E542K hyperactivating mutation, silent polymorphisms in *EGFR*, *H-RAS* and *TP53* with wild type status for R*ET*, *PTEN*, *CTNNB1*, *K-RAS*, *N-RAS*, *RAF-1* and *TRHB* [3-4, 13-14].

HTC/C3 were derived from cancer cells disseminated in the pleural fluid of a 44-year-old woman with a PTC-derived undifferentiated thyroid carcinoma highly resistant to chemotherapy [13]. They secrete IL1α, TGFα and PTH-like substance and have lost responsiveness to TSH; they have near-triploid karyotype with 23% polyploidy [13]. HTC/C3 express high levels of EGFR, they have heterozygous $BRAF^{V600E}$, TERT promoter pathogenic mutation, p53 p.P152L point mutation and wild type status of *RET, K-RAS, N-RAS* and *CTNNB1* [9, 14-15].

SW579 are derived from a squamous cell carcinoma of thyroid origin from a 59-year old caucasian male [16-17]. They have p53 p.I255S point mutation and wild type status of *BRAF*, *RET, K-RAS, N-RAS* and *CTNNB1* [14].

FRO are derived from a patient with a large cell undifferentiated thyroid carcinoma [18]. Although they have WT *TP53* they have a markedly decreased p53 mRNA content and do not express p53 at protein level [18-

19]. They express high levels of p27 and cyclin D3, intermediate to low levels of PTEN and harbour BRAF^{V600E} variant and CDKN2A c.238C>T nonsense mutation causing premature stop codon [15, 20-22]. SW1736 were established from an anaplastic thyroid cancer of a female patient. They have undetectable levels of TTF1 but high expression of several stem markers, like SOX2, OCT4, NANOG, C-MYC, SSEA4, and the ABCG2 transporter [23-24]. They harbour a heterozygous BRAF^{V600E} variant, TERT promoter pathogenic point mutation and have wild status for RET and PI3K [7, 9, 25-26]. Moreover they do not express p53 at protein level and express high levels of Δnp73α [20, 27-28].
Hth74 cells were established from an anaplastic thyroid carcinoma [29]. They express low levels of TSHR and have undetetable levels of TTF1 and PAX8 [5, 29-30]. They harbour TERT promoter mutation, p53 K286E point mutation, and overexpress TAp63α and TA/ΔNp73α [20, 27-28]; in addition they express low levels of PTEN [30-31]. Hht74 cells have silent polimorphisms in EGFR and TRHB genes and wild type status for RET CTNNB1, BRAF, H-RAS, K-RAS, N-RAS, RAF-1, PI3K [7, 9].

p53, BRAF and TERT sequencing

PCR amplification of exons 2-11 of the TP53 gene was performed with primer pairs described in the protocol of IARC (http://p53.iarc.fr/Download/TP53_DirectSequencing_IARC.pdf) as listed below.
Exons 2-3: forward TCTCATGCTGGATCCCCACT, reverse AGTCAGAGGACCAGGTCCTC
Exon 4: forward TGAGGACCTGGTCCTCTGAC, reverse AGAGGAATCCCAAAGTTCCA
Exons 5-6: forward TGTTCACTTGTGCCCTGACT, reverse TTAACCCCTCCTCCCAGAGA
Exon 7: forward CTTGCCACAGGTCTCCCCAA, reverse AGGGGTCAGAGGCAAGCAGA
Exon 8: forward TTCCTTACTGCCTCTTGCTT, reverse AGGCATAACTGCACCCTTGG
Exon 9: forward GACAAGAAGCGGTGGAG, reverse CGGCATTTTGAGTGTTAGAC
Exon 10: forward CAATTGTAACTTGAACCATC, reverse GGATGAGAATGGAATCCTAT
Exon 11: forward AGACCCTCTCACTCATGTGA, reverse TGACGCACACCTATTGCAAG
Additionally, an exon alternatively contained by some of the TP53 transcripts was also examined and PCR amplified with forward and reverse primeres 5'- CAATGGCTCCTGGTTGTAGC – 3' and 5'-AGCAGGCTAGGCTAAGCTATG – 3', respectively.
PCR amplification of TERT promoter and exon 15 of the BRAF gene were performed as previously reported [9, 32].
All of the PCR reactions were performed with GoTaq G2 Hot Start Polymerase (Promega) in a final volume of 20 µl, containing 50 ng genomic template DNA, 1,5 mM Mg2+, 0,2 mM of each of the 2'-deoxynucleoside 5'-triphosphates, 0,4 µM of the forward and the reverse primer as well, and 0.025 U/µl GoTaq G2 Hot Start Polymerase. PCR reaction conditions for exons 2-11 of the TP53 gene is described in the IARC protocol and "PCR program B" of the IARC protocol was used for the amplification of the alternatively used TP53 exon as well. PCR reaction condition used for the amplification of exon 15 of the BRAF gene was as follows: initial denaturation - 95 °C for two minutes; denaturation - 95 °C for 30 seconds, annealing - 54 °C for 30 seconds, elongation - 72 °C for 30 seconds. The amplification stage was repeated 35 times overall. Final elongation - 72 °C for 5 minutes.

PCR products were cleaned up with ExoSAP-IT (Affymetrix) according to the manufacturer’s protocol. Dye terminator cycle sequencing was performed with BigDye Terminator v3.1 Cycle Sequencing Kit (Life Technologies) according to the manufacturer’s instructions and, following gel filtration cleanup of the reaction products, sequencing was performed on Applied Biosystems 3500 Genetic Analyzer (Life Technologies).

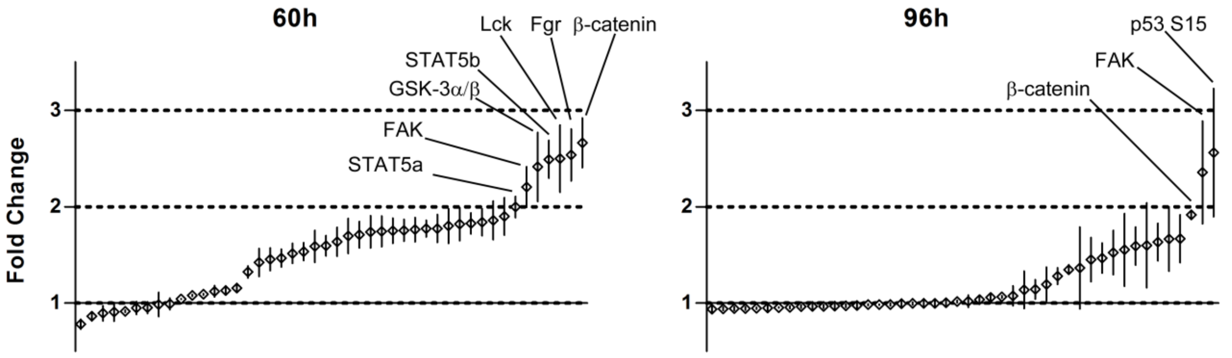

Supplemental Figure 1: human phospho-kinase antibody array.

Graphic representation of Human Phospho-Kinase Antibody Array. Kinase levels are plotted as fold change of 10 μM SP vs. DMSO treated HTC/C3 cells. Kinases with high significant changes ($p<0.01$ vs. reference control) are shown.

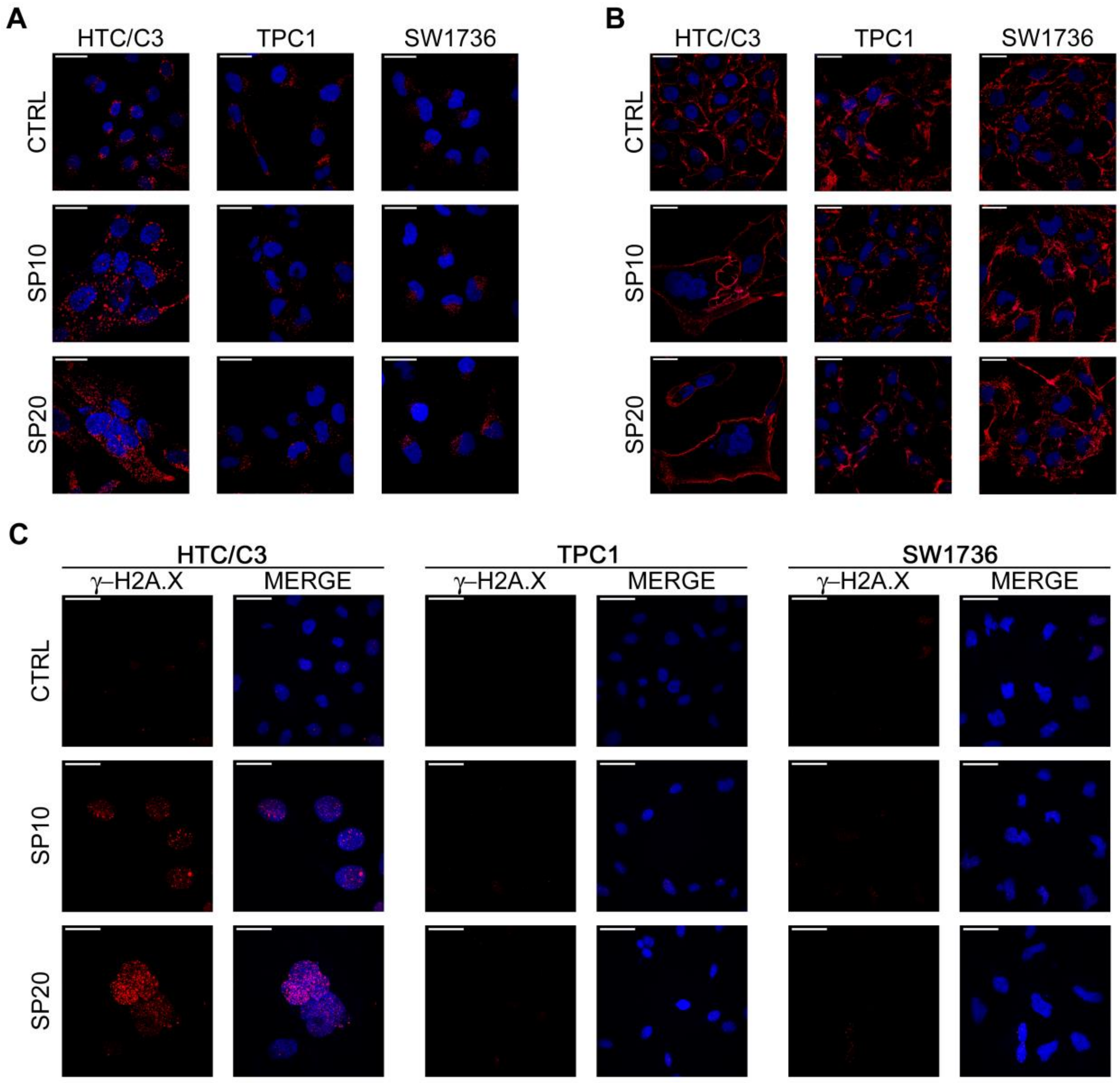

Supplemental Figure 2: additional senescence markers.

Whole-cell confocal microscopy images acquired after 10 μM SP (SP10), 20 μM SP (SP20) or equivalent amount of DMSO (CTRL) treatment for 96 hours. Scalebars: 30 μm. A) Lysosome (red) and nuclear (DAPI, blue) stainings. B) Membrane (red) and nuclear (DAPI, blue) stainings. C) γ-HA2.X (red) and nuclear (DAPI, blue) stainings.

Supplementary Table 1: SP activity in relation to thyroid cancer pathogenetic pathways.

Cell lines were grouped on the basis of literature reported abnormalities in p53 (WT: NTHY-ORI 3-1, TPC1 and K1; point mutations: HTC/C3, SW579 and Hth74; pseudo-null: SW1736 and FRO), BRAF (WT: NTHY-ORI 3-1, TPC1, SW579 and Hth74; V600E: HTC/C3, SW1736, K1 and FRO), PI3K/Akt pathway (WT: NTHY-ORI 3-1, HTC/C3, SW579 and SW1736; hyperactivated: TPC1, K1, Hth74 and FRO). Average values for growth inhibition at 10, 20 and 30 µM SP were extrapolated from each growth-inhibition curve with GraphPad Prism Software, version 5.04, and grouped accordingly to cell line status. **p<0.01, ***p<0.001 against respective WTs.

	**Status**	**SP 10µM**	**SP 20µM**	**SP 30µM**
**p53**	WT	84.16 ± 3.290	82.41 ± 3.556	76.64 ± 3.604
	Non functional	34.92 ± 6.065 ***	21.58 ± 4.124 ***	15.74 ± 3.188 ***
	Pseudo null	78.42 ± 1.885	62.80 ± 1.957 **	52.95 ± 3.682 ***
**BRAF**	WT	67.51 ± 5.049	59.70 ± 7.026	51.73 ± 8.579
	V600E	67.47 ± 8.494	57.83 ± 8.384	49.06 ± 6.840
**PI3K/Akt**	WT	60.57 ± 6.545	50.03 ± 6.212	44.18 ± 6.173
	Hyperactivation	77.30 ± 6.069	71.42 ± 9.306	60.76 ± 10.14

Supplementary Table 2: Human Phospho-Kinase Antibody Array.

Human Phospho-Kinase Antibody Array data expressed as fold change of 10 μM vs. DMSO treated HTC/C3 cells. Results are reported as mean ± SEM. *p<0.05, **p<0.01 and ***p<0.001 vs. reference control.

	**60 hours**	**96 hours**
Akt S473	1.74 ± 0.31	0.97 ± 0.03
Akt T308	1.09 ± 0.01	1.37 ± 0.85
AMPKα1 T184	1.90 ± 0.39	1.02 ± 0.03
AMPKα2 T172	1.77 ± 0.29	0.98 ± 0.02
β-catenin	2.66 ± 0.51 ***	1.91 ± 0.06 **
Chk-2 T68	1.64 ± 0.30	0.94 ± 0.04
c-Jun S63	0.90 ± 0.04	1.14 ± 0.39
CREB S133	0.99 ± 0.25	0.95 ± 0.01
EGFR Y1086	1.70 ± 0.36	0.96 ± 0.04
eNOS S1177	0.87 ± 0.09	1.60 ± 0.88
ERK 1/2 T202/Y204. T185/Y187	2.17 ± 0.70	0.95 ± 0.02
FAK Y397	1.86 ± 0.40 ***	2.36 ± 1.07 ***
Fgr Y412	2.20 ± 0.42 ***	1.67 ± 0.67
Fyn Y420	1.53 ± 0.18	0.99 ± 0.09
GSK-3α/β S21/S9	1.83 ± 0.22 ***	0.94 ± 0.04
Hck Y411	1.71 ± 0.27	1.02 ± 0.13
HSP27 S78/S82	1.74 ± 0.33	0.97 ± 0.04
HSP60	1.08 ± 0.02	1.28 ± 0.17
JNK pan T183/Y185. T221/Y223	1.80 ± 0.36	1.00 ± 0.07
Lck Y394	2.49 ± 0.39 ***	1.63 ± 0.39
Lyn Y397	1.52 ± 0.21	0.99 ± 0.03
MSK1/2 S376/S360	2.00 ± 0.22 *	0.95 ± 0.05
p27 T198	0.95 ± 0.12	1.03 ± 0.08
p38α T180/Y182	1.84 ± 0.28	0.96 ± 0.08
p53 S15	1.32 ± 0.12	2.56 ± 1.33 ***
p53 S392	0.99 ± 0.12	1.14 ± 0.20
p53 S46	1.16 ± 0.07	1.20 ± 0.35
p70 S6 K T389	1.13 ± 0.09	1.00 ± 0.05

p70 S6 K T421/ S424	1.59 ± 0.33	1.06 ± 0.03
PDGF Rβ Y751	1.82 ± 0.34	1.06 ± 0.09
PLC-γ1 Y783	0.89 ± 0.16	1.45 ± 0.46
PRAS40 T246	1.47 ± 0.18	0.94 ± 0.03
PYK2 Y402	0.95 ± 0.13	1.67 ± 0.50
reference	1.04 ± 0.01	0.98 ± 0.06
RSK 1/2/3 S380/S386/S377	0.91 ± 0.19	1.55 ± 0.75
Src Y419	1.46 ± 0.23	1.59 ± 0.39
STAT2 Y689	1.75 ± 0.26	0.94 ± 0.05
STAT3 S727	0.92 ± 0.02	1.47 ± 0.31
STAT3 Y705	1.60 ± 0.21	1.52 ± 0.46
STAT5a Y694	2.50 ± 0.70 **	1.00 ± 0.05
STAT5a/b Y694/Y699	1.77 ± 0.17	0.97 ± 0.06
STAT5b Y699	2.54 ± 0.54 ***	1.00 ± 0.03
STAT6 Y641	1.76 ± 0.25	0.98 ± 0.01
TOR S2448	1.75 ± 0.23	1.07 ± 0.21
WNK1 Y402	0.78 ± 0.10	1.35 ± 0.10
Yes Y426	1.42 ± 0.29	0.96 ± 0.03

Supplemental material references

1. Lemoine NR, Mayall ES, Jones T, Sheer D, McDermid S, Kendall-Taylor P, Wynford-Thomas, D. Characterisation of human thyroid epithelial cells immortalised in vitro by simian virus 40 DNA transfection. *Br J Cancer.* 1989; 60(6):897-903.
2. Maric I, Viaggi S, Caria P, Frau DV, Degan P, Vanni R. Centrosomal and mitotic abnormalities in cell lines derived from papillary thyroid cancer harboring specific gene alterations. *Mol Cytogenet.* 2011; 4:26.
3. Ishizaka Y, Itoh F, Tahira T, Ikeda I, Ogura T, Sugimura T, Nagao, M. Presence of aberrant transcripts of ret proto-oncogene in a human papillary thyroid carcinoma cell line. *Jpn J Cancer Res.* 1989; 80(12):1149-52.
4. van Staveren WC, Solis DW, Delys L, Duprez L, Andry G, Franc B, Thomas G, Libert F, Dumont JE, Detours V, Maenhaut C. Human thyroid tumor cell lines derived from different tumor types present a common dedifferentiated phenotype. *Cancer Res.* 2007; 67(17):8113-20.
5. Schweppe RE, Klopper JP, Korch C, Pugazhenthi U, Benezra M, Knauf JA, Fagin JA, Marlow LA, Copland JA, Smallridge RC, Haugen BR. Deoxyribonucleic acid profiling analysis of 40 human thyroid cancer cell lines reveals cross-contamination resulting in cell line redundancy and misidentification. *J Clin Endocrinol Metab.* 2008; 93(11):4331-41.
6. Saiselet M, Floor S, Tarabichi M, Dom G, Hebrant A, van Staveren WC, Maenhaut C. Thyroid cancer cell lines: an overview. *Front Endocrinol (Lausanne).* 2012; 3:133.
7. Meireles AM, Preto A, Rocha AS, Rebocho AP, Maximo V, Pereira-Castro I, Moreira S, Feijao T, Botelho T, Marques R, Trovisco V, Cirnes L, Alves C, Velho S, Soares P, Sobrinho-Simoes M. Molecular and genotypic characterization of human thyroid follicular cell carcinoma-derived cell lines. *Thyroid.* 2007; 17(8):707-15.
8. Jossart GH, Greulich KM, Siperstein AE, Duh Q, Clark OH, Weier HU. Molecular and cytogenetic characterization of a t(1;10;21) translocation in the human papillary thyroid cancer cell line TPC-1 expressing the ret/H4 chimeric transcript. *Surgery.* 1995; 118(6):1018-23.
9. Landa I, Ganly I, Chan TA, Mitsutake N, Matsuse M, Ibrahimpasic T, Ghossein RA, Fagin JA. Frequent somatic TERT promoter mutations in thyroid cancer: higher prevalence in advanced forms of the disease. *J Clin Endocrinol Metab.* 2013; 98(9):E1562-6.
10. Ribeiro FR, Meireles AM, Rocha AS, Teixeira MR. Conventional and molecular cytogenetics of human non-medullary thyroid carcinoma: characterization of eight cell line models and review of the literature on clinical samples. *BMC Cancer.* 2008; 8:371.
11. Antonini P, Linares G, Gaillard N, Venuat AM, Schlumberger M, Travagli JP, Caillou B, Berger R, Parmentier C. Cytogenetic characterization of a new human papillary thyroid carcinoma permanent cell line (GLAG-66). *Cancer Genet Cytogenet.* 1993; 67(2):117-22.
12. Challeton C, Branea F, Schlumberger M, Gaillard N, de Vathaire F, Badie C, Antonini P, Parmentier C. Characterization and radiosensitivity at high or low dose rate of four cell lines derived from human thyroid tumors. *Int J Radiat Oncol Biol Phys.* 1997; 37(1):163-9.
13. Enomoto T, Sugawa H, Inoue D, Miyamoto M, Kosugi S, Takahashi T, Kitamura N, Yamamoto I, Konishi J, Mori T, Imura H. Establishment of a human undifferentiated thyroid cancer cell line producing several growth factors and cytokines. *Cancer.* 1990; 65(9):1971-9.
14. Yoshimoto K, Iwahana H, Fukuda A, Sano T, Saito S, Itakura M. Role of p53 mutations in endocrine tumorigenesis: mutation detection by polymerase chain reaction-single strand conformation polymorphism. *Cancer* . 1992; 52(18):5061-4.
15. Zou M, Baitei EY, Alzahrani AS, BinHumaid FS, Alkhafaji D, Al-Rijjal RA, Meyer BF, Shi Y. Concomitant RAS, RET/PTC, or BRAF mutations in advanced stage of papillary thyroid carcinoma. *Thyroid.* 2014; 24(8):1256-66.
16. Fogh J, Fogh JM, Orfeo T. One hundred and twenty-seven cultured human tumor cell lines producing tumors in nude mice. *J Natl Cancer Inst.* 1977; 59(1):221-6.
17. Fogh J, Wright WC, Loveless JD. Absence of HeLa cell contamination in 169 cell lines derived

from human tumors. *J Natl Cancer Inst.* 1977; 58(2):209-14.
18. Fagin JA, Matsuo K, Karmakar A, Chen DL, Tang SH, Koeffler HP. High prevalence of mutations of the p53 gene in poorly differentiated human thyroid carcinomas. *J Clin Invest.* 1993; 91(1):179-84.
19. Namba H, Hara T, Tukazaki T, Migita K, Ishikawa N, Ito K, Nagataki S, Yamashita S. Radiation-induced G1 arrest is selectively mediated by the p53-WAF1/Cip1 pathway in human thyroid cells. *Cancer Res.* 1995; 55(10):2075-80.
20. Malaguarnera R, Mandarino A, Mazzon E, Vella V, Gangemi P, Vancheri C, Vigneri P, Aloisi A, Vigneri R, Frasca F. The p53-homologue p63 may promote thyroid cancer progression. *Endocr Relat Cancer.* 2005; 12(4):953-71.
21. Mitsiades CS, Negri J, McMullan C, McMillin DW, Sozopoulos E, Fanourakis G, Voutsinas G, Tseleni-Balafouta S, Poulaki V, Batt D, Mitsiades N. Targeting BRAFV600E in thyroid carcinoma: therapeutic implications. *Mol Cancer Ther.* 2007; 6(3):1070-8.
22. Calabro V, Strazzullo M, La Mantia G, Fedele M, Paulin C, Fusco A, Lania L. Status and expression of the p16INK4 gene in human thyroid tumors and thyroid-tumor cell lines. *Int J Cancer.* 1996; 67(1):29-34.
23. Heldin NE, Westermark B. The molecular biology of the human anaplastic thyroid carcinoma cell. *Thyroidology.* 1991; 3(3):127-31.
24. Carina V, Zito G, Pizzolanti G, Richiusa P, Criscimanna A, Rodolico V, Tomasello L, Pitrone M, Arancio W, Giordano C. Multiple pluripotent stem cell markers in human anaplastic thyroid cancer: the putative upstream role of SOX2. *Thyroid.* 2013; 23(7):829-37.
25. Xu X, Quiros RM, Gattuso P, Ain KB, Prinz RA. High prevalence of BRAF gene mutation in papillary thyroid carcinomas and thyroid tumor cell lines. *Cancer Res.* 2003; 63(15):4561-7.
26. Liu R, Liu D, Trink E, Bojdani E, Ning G, Xing M. The Akt-specific inhibitor MK2206 selectively inhibits thyroid cancer cells harboring mutations that can activate the PI3K/Akt pathway. *J Clin Endocrinol Metab.* 2011; 96(4):E577-85.
27. Messina RL, Sanfilippo M, Vella V, Pandini G, Vigneri P, Nicolosi ML, Giani F, Vigneri R, Frasca F. Reactivation of p53 mutants by prima-1 [corrected] in thyroid cancer cells. *Int J Cancer.* 2012; 130(10):2259-70.
28. Frasca F, Vella V, Aloisi A, Mandarino A, Mazzon E, Vigneri R, Vigneri P. p73 tumor-suppressor activity is impaired in human thyroid cancer. *Cancer Res.* 2003; 63(18):5829-37.
29. Heldin NE, Cvejic D, Smeds S, Westermark B. Coexpression of functionally active receptors for thyrotropin and platelet-derived growth factor in human thyroid carcinoma cells. *Endocrinology.* 1991; 129(4):2187-93.
30. Pilli T, Prasad KV, Jayarama S, Pacini F, Prabhakar BS. Potential utility and limitations of thyroid cancer cell lines as models for studying thyroid cancer. *Thyroid.* 2009; 19(12):1333-42.
31. Lee JJ, Foukakis T, Hashemi J, Grimelius L, Heldin NE, Wallin G, Rudduck C, Lui WO, Hoog A, Larsson C. Molecular cytogenetic profiles of novel and established human anaplastic thyroid carcinoma models. *Thyroid.* 2007; 17(4):289-301.
32. Spittle C, Ward MR, Nathanson KL, Gimotty PA, Rappaport E, Brose MS, Medina A ,Letrero R., Herlyn M, Edwards RH. Application of a BRAF pyrosequencing assay for mutation detection and copy number analysis in malignant melanoma. *J Mol Diagn.* 2007; 9(4):464-71.
